# Implant Choice and Outcomes of the Sinus Tarsi Approach for Displaced Intra-articular Calcaneal Fractures

**DOI:** 10.1177/10711007231176276

**Published:** 2023-05-30

**Authors:** Robin Eelsing, Loran B. Aronius, Jens A. Halm, Tim Schepers

**Affiliations:** 1 Department of Surgery, Amsterdam UMC location University of Amsterdam, Meibergdreef 9, Amsterdam, the Netherlands; 2 Amsterdam Movement Sciences, AMS - Musculoskeletal Health, Amsterdam, the Netherlands

**Keywords:** calcaneal fracture, sinus tarsi approach, screw fixation, plate fixation, anatomic plate fixation

## Abstract

**Background:**

Operative fixation of displaced intra-articular calcaneal fractures is considered the gold standard, for which multiple fixation methods are available. This study compares the (functional) outcome of screw fixation (SF), plate fixation (PF), and anatomical plate fixation (APF) via the sinus tarsi approach (STA).

**Methods:**

A total of 239 patients (265 fractured calcanei) who received surgical treatment of a displaced intra-articular calcaneal fracture via STA between 2011 and 2022 were included.

**Results:**

Böhler angle (BA) measured immediately postoperatively (BA post-OR) and the decrease in BA at 1 year (∆BA) differed significantly in favor of PF/APF compared with SF (BA post-OR: SF vs PF *P* = .010 and SF vs APF *P* = .001; ∆BA: SF vs PF *P* = .032 and SF vs APF *P* = .042). Implant removal surgery was performed significantly less in the APF group as compared to the SF/PF groups (APF vs SF/PF; 9.9% vs 22.9%/23.7%, *P* = .015). Surgical site infections and secondary arthrodesis of the subtalar joint occurred equally in the 3 groups. Furthermore, the mean American Orthopaedic Foot & Ankle Society ankle-hindfoot scale, Foot Function Index score, and EuroQOL-5D-index / visual analog scale score, did not differ notably between SF, PF, and APF.

**Conclusion:**

The results show that both PF and APF are favored over SF because of an improved correction of BA measured directly postoperatively, a lower secondary loss of BA and, for APF, a lower implant removal rate. There was no difference in the rate of surgical site infections, need for secondary arthrodesis, nor functional outcome scores between different implants using the STA.

**Level of Evidence:**

Level III, retrospective cohort study.

## Introduction

Although an (inter)national algorithm for the surgical treatment of calcaneal fractures is still lacking, treatment options for calcaneal fractures have been widely described. For displaced intra-articular calcaneal fractures (DIACFs), operative fixation is considered the gold standard. The operative management of calcaneal fractures can be divided into 2 different groups: the open reduction internal fixation (ORIF) and percutaneous reduction internal fixation (PRIF). For ORIF different approaches are available, such as the extended lateral approach (ELA) and the sinus tarsi approach (STA), with each approach having its own advantages and disadvantages.

In the last decade, for many indications, STA has proven to itself as an equal or even superior alternative to ELA. A postoperative wound complication rate of 5% via STA is described, which is significantly better than the 20% or more reported described for surgery performed via an ELA.^9,10,17,18^ In addition, the quality of the reconstruction via STA has proven to be similar to that of the calcaneal reconstructions performed through ELA.^11,21,22^

Utilizing STA, different fixation methods can be used such as screw fixation (SF), plate fixation (PF), and more recently anatomic plate fixation (APF) specifically designed for the calcaneus and indicated for the sinus tarsi approach. Although there is literature available comparing the outcome of both SF and PF or APF,^3,5,7,12,16^ there is a paucity comparing the surgical outcome of SF, PF, and APF while combining this with functional and quality of life patient-reported outcomes. Therefore, the aim of this study is to compare both the surgical, functional, and quality of life outcome of SF, PF, and APF via STA for DIACFs.

## Materials and Methods

This study was granted a waiver waived by the Medical Research Ethics Committee of our hospital. Data were acquired from a database consisting of 311 consecutive patients who underwent surgical treatment via STA for a DIACF between August 2012 and January 2022. Included patients were aged 16 years or older. Patients who underwent a primary arthrodesis of the subtalar joint were excluded. After a patient was deemed eligible, written informed consent was obtained in accordance with the World Medical Association Declaration of Helsinki.

### Data Collection

The following patient characteristics were collected: age, gender, affected side, smoking, length, weight, and a history of diabetes mellitus. In addition to this, fracture characteristics such as the Böhler angle (BA),^1^ Essex-Lopresti classification,^4^ and Sanders classification^14^ were determined.

BA was measured on a lateral radiograph at presentation, directly postsurgery, at 6 months and 1 year. Fracture classification and angle measurements were performed once by one of the 3 authors on the available radiographs and computed tomographic imaging. When a measurement was deemed inconclusive by one of the raters, it was discussed with the other raters in a consensus meeting and consensus was reached. Further data were collected retrospectively from the medical records in December 2022.

From 2014 onward, paper surveys were sent to the patients at approximately 18 months’ follow-up. In March 2022, an equivalent survey was sent using Castor EDC (Castor, Amsterdam, North Holland, the Netherlands) to all patients who had a valid email address, including those who had already received a paper survey. The survey consisted of 4 different questionnaires: the American Orthopaedic Foot & Ankle Society ankle-hindfoot scale (AOFAS),^8^ Foot Function Index (FFI),^2^ EuroQOL-5D-3L (EQ5D-3L),^13^ and EuroQOL-5D-5L (EQ5D-5L).^6^

### Surgical Technique

The patient is positioned on a beanbag in a lateral decubitus position on the contralateral side of the injury. The injured leg is flexed at the knee and hip, and the contralateral leg is in a straight position. The beanbag is raised at the level of the ankle. As it supports the ankle, the foot is free, which allows for temporarily adding varus of the hindfoot during the procedure to visualize the subtalar joint.

The STA is made from 5 mm below the tip of the distal fibula toward the base of the fourth metatarsal. The incision is usually between 3 and 4 cm in length and can be extended in the case of anterior process and/or calcaneocuboid joint involvement. The peroneal tendon sheath is preserved as much as possible, and tendons are held plantar-ward. The sural nerve is not routinely explored, but if encountered, it is freed up and protected. Following debridement, the subtalar joint and the crucial angle of Gissane are visualized. A subperiosteal lateral flap is created using a broad periosteal elevator and a No. 15 blade. Depending on the fracture type, different reduction techniques are used, which have been described previously.^19^

**Figure 1.**
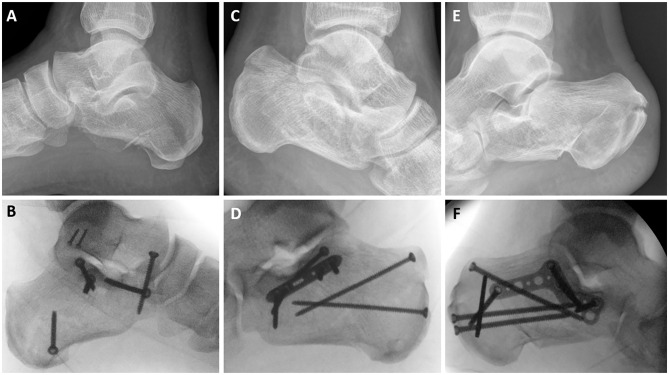
Different fixation methods and the concordant prereconstructive imaging. (A, B) Screw fixation for DIACF. (C, D) Plate fixation for DIACF. (E, F) Anatomic plate fixation for DIACF. DIACF, displaced intra-articular calcaneal fracture.

Figure 1 displays the different fixation methods. Screw-only reconstructions (Figure 1B) were included in the SF group. Reconstructions fixated with generic plating (Figure 1D) were included in the PF group. In case plating specifically designed for fixating calcaneal fractures (MINI-Calc Plate; Acumed, Hillsboro, OR) (Figure 1F) was used for the reconstruction, they were included in the APF group. One trauma surgeon specialized in foot/ankle surgery was present during all operations, both as the main surgeon or as the assistant surgeon. The decision for the type of fixation was made at the time of the surgery based on the preferences of this surgeon to the specific patient, soft tissue conditions, and fracture pattern.

### Outcomes and Statistical Analysis

The primary outcomes were BA immediately postoperatively (BA post-OR) and decrease in BA after 1 year as compared to BA post-OR (∆BA).

Secondary outcomes were surgical site infections (SSIs), secondary subtalar arthrodesis (SAD), implant removal, failed fixation, and patient-reported outcomes: AOFAS score, FFI score and EQ5D-index/VAS. Failed fixation was defined as a decrease in BA ≥10 degrees within 1 year (∆BA ≥ 10).

Data were analyzed using the IBM Statistical Package for the Social Sciences (SPSS) version 26.0 (IBM; Armonk, NY, USA). When the test conditions were met, a chi-squared test, Fisher exact test, or Kruskal-Wallis 1-way analysis of variance was run to describe intergroup variability. A generalized linear model (GLM) was run to analyze continuous outcomes, which made it possible to adjust for significant differences in outcome predicting variables between groups. To overcome the problem of type I errors due to multiple testing, *P* values for the patient-reported outcomes were omitted. At last, a post hoc power analysis was performed.

## Results

**Figure 2.**
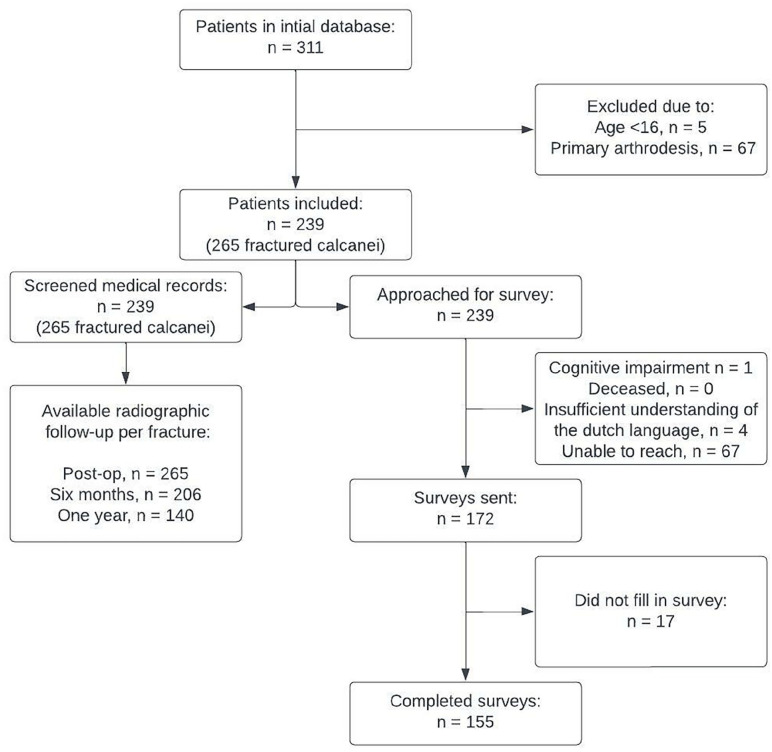
Flow diagram of follow-up.

Patient characteristics are displayed in Table 1. A total of 72 patients met the exclusion criteria, resulting in the inclusion of 239 patients with 265 fractured calcanei. The median follow-up of the surveys was 45.2 months, and 155 patients fully completed the survey. A flow chart of the follow-up is shown in Figure 2.

**Table 1.** Characteristics.

	Total, n [no. of feet] = 239 [265]	SF, n [no. of feet] = 80 [83]	PF, n [no. of feet] = 56 [59]	APF, n [no. of feet] = 108 [123]	*P* Value^a^
Male gender, n [no. of feet] (%)	182 [200] (76.2)	55 [57] (68.8)	42 [43] (75.0)	87 [100] (80.6)	.103^b^
Age, median (IQR)	44 (23)	41 (20)	42 (24)	45 (24)	.987^c^
BMI, median (IQR)	23.31 (4.57)	24.07 (4.97)	22.91 (5.33)	23.72 (4.16)	.380^c^
ASA class, n (%)	265 (100)	83 (100)	59 (100)	123 (100)	.357^b^
ASA 1	172 (64.9)	59 (71.1)	36 (61.0)	77 (62.6)	
ASA 2	85 (32.1)	23 (27.7)	22 (37.3)	40 (32.5)	
ASA 3	8 (3.0)	1 (1.2)	1 (1.7)	6 (4.9)	
Sanders, n (%)	264 (99.6)	82 (98.8)	59 (100)	123 (100)	**.008** ^b^
I	3 (1.1)	0 (0.0)	2 (3.4)	1 (0.8)	
II	177 (66.8)	64 (77.1)	42 (71.2)	71 (57.7)	
III	70 (26.4)	16 (19.3)	10 (16.9)	44 (35.8)	
IV	14 (5.3)	2 (2.4)	5 (8.5)	7 (5.7)	
Essex-Lopresti, n (%)	264 (99.6)	82 (98.8)	59 (100)	123 (100)	**.009** ^b^
Joint depression	111 (41.9)	27 (32.5)	20 (33.9)	64 (52.0)	
Tongue	153 (57.7)	55 (66.3)	39 (66.1)	59 (48.0)	
Mean Böhler angle preoperation, degrees (SD)	-2.75 (15.07)	-0.56 (14.80)	-1.14 (15.06)	-5.00 (15.07)	.077^c^
Open fracture, n (%)	4 (1.5)	1 (1.2)	0 (0.0)	3 (2.4)	.690^d^
Gustillo I	4 (100)	1 (100)	0 (0.0)	3 (100)	
Survey follow-up, mo, median (IQR)	45.17 (54.02)	66.51 (60.07)	82.82 (36.53)	29.75 (27.73)	**<.001** ^b^

Abbreviations: APF, anatomical plate fixation; ASA, American Society of Anesthesiologists; BMI, body mass index; IQR, interquartile range; PF, plate fixation; SF, screw fixation.

a Significant *P* values are bold.

b Chi-squared test.

c Kruskal-Wallis 1-way analysis of variance.

d Fisher exact test.

In Table 2 the adjusted results of BA postoperatively, ∆BA, SSI, SAD, implant removal, and failed fixation are displayed. The data was adjusted for BA preoperatively, Sanders classification, and Essex-Lopresti classification. Significant differences in favor of PF and APF were seen when compared to SF for BA postoperatively and ∆BA. The frequency of SSI and SAD did not differ significantly between SF, PF, and APF (*P* = .352 and *P* = .852). Implant removal was performed significantly less in the APF group when compared to SF/PF (*P* = .015). Mean duration until implant removal (SF 56.9, PF 71.9, and APF 31.4 months) did not differ significantly between the groups (*P* = .171). Even though SF had a 2 to almost 3 times higher rate of failed fixation compared to the other groups, the amount of failed fixations did not differ significantly between groups (*P* = .251), given the current number of patients.

**Table 2.** Surgical Outcomes.

Outcome	Adjusted Mean (95% CI) or n (%)	Adjusted *P* Value^a^
BA post-OR in degrees		
SF vs PF	27.36 (26.17-28.55) vs 29.75 (28.36-31.14)	**.010** ^b^
SF vs APF	27.36 (26.17-28.55) vs 30.45 (29.47-31.43)	**<.001** ^b^
PF vs APF	29.75 (28.36-31.14) vs 30.45 (29.47-31.43)	.423^b^
∆BA, degrees		
SF vs PF	5.78 (4.61-6.95) vs 3.92 (2.67-5.16)	**.032** ^b^
SF vs APF	5.78 (4.61-6.95) vs 4.12 (3.04-5.19)	**.042** ^b^
PF vs APF	3.92 (2.67-5.16) vs 4.12 (3.04-5.19)	.815^b^
Surgical site infection	13 (4.9)	.352^c^
SF	3 (3.6)	
PF	5 (8.5)	
APF	5 (4.1)	
Secondary subtalar arthrodesis	17 (6.4)	.852^c^
SF	6 (7.2)	
PF	4 (6.8)	
APF	7 (5.7)	
Implant removal	45 (17.0)	**.015** ^c^
SF	19 (22.9)	
PF	14 (23.7)	
APF	12 (9.9)	
Failed fixation	13 (5.9)	.251^c^
SF	7 (9.9)	
PF	2 (3.7)	
APF	4 (4.2)	

Abbreviations: APF, anatomical plate fixation; BA post-OR, Böhler angle measured immediately postoperatively; ∆BA, decrease in BA after 1 year as compared to BA post-OR; PF, plate fixation; SF, screw fixation.

a Significant *P* values are bold.

b Generalized linear model.

c Fisher exact test.

AOFAS, FFI, and EQ5D scores are displayed in Table 3. The data were adjusted for BA preoperatively, Sanders classification, Essex-Lopresti classification, and the length of follow-up. Mean AOFAS, FFI, and EQ5D scores were similar between the 3 groups. Additionally, after exclusion of bilateral fractures, BA at presentation, BA post-OR, and ∆BA did not predict outcome based on the AOFAS (*P* = .788, *P* = .547, and *P* = .992), FFI (*P* = .441, *P* = .567, and *P* = .659), and EQ5D (*P* = .853, *P* = .940, and *P* = .928) scores.

**Table 3.** Patient-Reported Outcomes.

Outcome	Adjusted Mean (95% CI)
AOFAS	
SF	77.54 (71.81-83.26)
PF	77.27 (70.40-84.14)
APF	76.63 (71.77-81.48)
FFI	
SF	20.60 (14.22-26.98)
PF	20.52 (12.68-28.37)
APF	23.03 (17.51-28.55)
EQ5D-index	
SF	0.803 (0.741-0.865)
PF	0.829 (0.753-0.905)
APF	0.787 (0.734-0.841)
EQ5D/VAS	
SF	81.30 (76.89-85.71)
PF	80.63 (75.20-86.05)
APF	81.28 (77.46-85.09)

Abbreviations: AOFAS, American Orthopaedic Foot & Ankle Society ankle-hindfoot scale; APF, anatomical plate fixation; EQ5D, EuroQOL-5D; FFI, Foot Function Index; PF, plate fixation; SF, screw fixation; VAS, visual analog scale.

Post hoc power analysis revealed learned that considering the common SD and primary outcomes (BA post-OR/∆BA), a minimum of 49 patients was necessary for BA post-OR and a minimum of 65 patients for ∆BA in order to have at least 80% power.

## Discussion and Conclusion

Patient-reported outcomes (AOFAS, FFI, and EQ5D), number of SSIs, SAD, and failed fixation were comparable between the 3 groups. A significant difference was seen in the BA post-OR, ∆BA, and number of implant removal procedures.

As mentioned earlier, literature comparing SF, PF, and APF for DIACFs via STA is currently not available. However, studies comparing SF with nonspecified PF^12^ and SF with APF^3,5,7^ are available for comparison.

Unlike the results of our analysis, Pitts et al,^12^ who compared SF with nonspecified PF, found no significant differences in BA postoperatively, decrease in BA after approximately 1 year, and SSI between SF and PF/APF. On the other hand, Guo et al^5^ and Sato et al^16^ described results comparable to our study, despite a significantly higher implant removal rate for APF as compared to SF in the study of Guo et al.^5^ One of the possible explanations for the higher implant removal rate could be the longer follow-up (mean 44.2 months) for the APF group as compared to our study (median 29.8 months). In addition to this, Chotikkakamthorn et al^3^ showed that SF, in comparison to APF, provided inferior stability of the posterior facet fixation and calcaneal varus reduction with a lower complication rate. Finally, Kir et al^7^ described a significant difference in the amount of reinterventions, complex regional pain syndrome, and Maryland Foot Score in favor of APF when compared to SF.

Although the recent systematic review and meta-analysis of Wang et al^20^ concluded that SF is favored over PF, it has to be kept in mind that their study combined both radiologic and functional outcome of (randomized) prospective studies and retrospective studies while not mentioning the surgical approach and potential selection bias.

An interesting observation is that the preoperative BA was significantly lower and the Sanders classification significantly higher in the APF group when compared to the SF and PF groups. This suggests that in more complex injuries, treatment with APF was favored over SF and PF. As more complex injuries are associated with a poorer outcome,^14,15^ significant differences between the groups in favor of SF and PF were expected. Despite these expectations, the opposite was seen. The results of APF were, if not favorable, at least comparable to SF and PF. Interestingly, after exclusion of bilateral fractures, BA at presentation, BA post-OR, and ∆BA did not predict outcome for the overall population based on the AOFAS (*P* = .788, *P* = .547, and *P* = .992), FFI (*P* = .441, *P* = .567, and *P* = .659), and EQ5D (*P* = .853, *P* = .940, and *P* = .928) scores debating the clinical importance of BA at the different stages of treatment.

Another consequence of the heterogeneity between the groups, more specifically SF and PF being performed more often in less complex injuries, is that SF and PF might falsely be considered as reliable fixation methods because of selection bias. Additionally, post hoc power analysis showed that the study is underpowered in regard to the PF. The importance is debatable, as the results show clear significant differences with SF.

As APF was introduced later on the market than the other implants, the follow-up was significantly lower in the APF group when compared to both the SF and PF groups. This did not affect the primary outcome of ∆BA although it might have affected the other outcomes such as the patient-reported outcomes, amount of SAD, and implant removal. Furthermore, the improvement in skills of the surgeon performing the surgeries might have influenced the outcome as APF was introduced later and the baseline skill of the surgeon might have been higher when compared to the other groups. Additionally, as mentioned in the Results section, the time after which implant removal was performed did not significantly differ between the groups. However, big differences in the mean duration after which implant removal was performed are seen, not taking away the risk of bias due to the difference in length of follow-up for the implant removal outcome.

In our opinion, the results of this study can be considered reliable because of the large cohort, simultaneous comparison of the 3 main fixation techniques, homogeneity in surgeons performing the surgeries, and length of follow-up. The main limitations were the heterogeneity between groups and the retrospective design of the study. Finally, as it could have led to bias, the unavertable loss to follow-up of 35% of patient-reported outcomes has to be noted.

In conclusion, for reconstruction via the STA, (anatomic) PF is favored over SF due to the higher Böhler angle measured directly postoperatively, the lower secondary loss of Böhler angle and, for APF, a lower implant removal rate while not affecting the rate of SSIs, need for secondary arthrodesis, number of failed fixations, and functional outcome scores. However, the differences between the groups were small, indicating that probably more important in dictating outcome is the severity of the initial injury and the quality of reduction, rather than the type of implant.

## Supplemental Material

sj-pdf-1-fai-10.1177_10711007231176276 – Supplemental material for Implant Choice and Outcomes of the Sinus Tarsi Approach for Displaced Intra-articular Calcaneal Fractures Click here for additional data file. Supplemental material, sj-pdf-1-fai-10.1177_10711007231176276 for Implant Choice and Outcomes of the Sinus Tarsi Approach for Displaced Intra-articular Calcaneal Fractures by Robin Eelsing, Loran B. Aronius, Jens A. Halm and Tim Schepers in Foot & Ankle International
